# Macroglossia and lip edema: A case of paraproteinemia-associated scleredema responsive to intravenous immunoglobulins

**DOI:** 10.1016/j.jdcr.2023.11.033

**Published:** 2023-12-27

**Authors:** Onjona B. Hossain, Daniel R. Antohi, Tian Ran Zhu, Michael Occidental, Amin Bijal, Benedict Wu

**Affiliations:** a Division of Dermatology, Montefiore Einstein, Albert Einstein College of Medicine, Bronx, New York; b Department of Pathology, Montefiore Einstein, Albert Einstein College of Medicine, Bronx, New York

**Keywords:** atypical scleredema presentation, dermatopathology, intravenous immunoglobulin, monoclonal gammopathy, scleredema adultorum Buschke

## Introduction

Scleroedema adultorum Buschke (scleredema) is a diffuse, symmetrical, progressive, nonpitting skin induration characterized by increased collagen and mucin deposition in the dermis.^1^ Treatment for scleredema is limited due to the rarity of this condition. We present a case of scleredema with the unique clinical findings of macroglossia and lip edema and report intravenous immunoglobulin (IVIg) as a treatment option in paraproteinemia-associated scleredema (PAS).

## Case report

**Fig 1:**
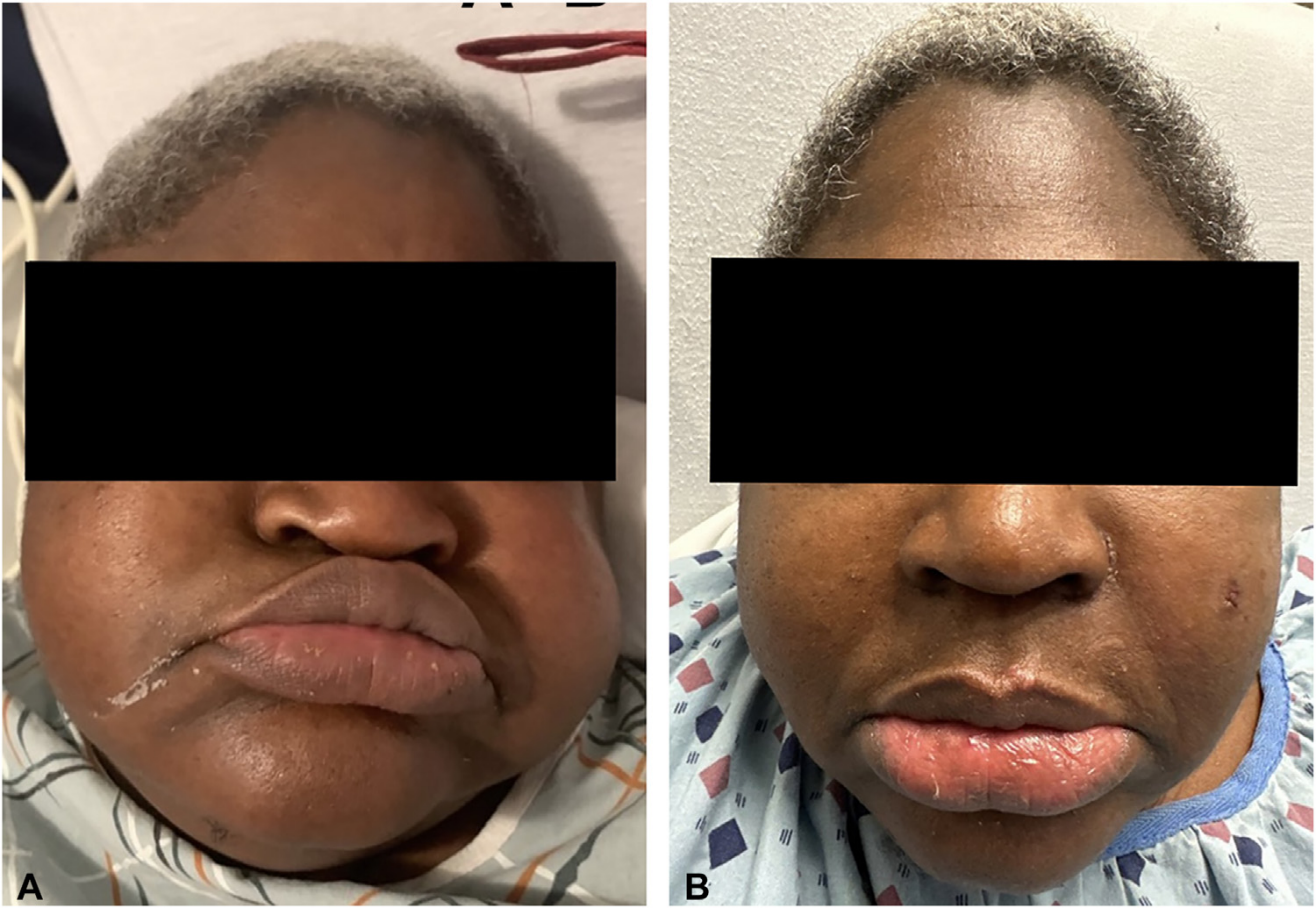
Improvement of lip and cheek edema with intravenous immunoglobulin therapy. **A,** Initial presentation showing marked skin induration and edema affecting the cheeks and lips with loss of nasolabial folds. **B,** Decreased facial and lip edema following the first course of intravenous immunoglobulin with the return of nasolabial folds.

**Fig 2:**
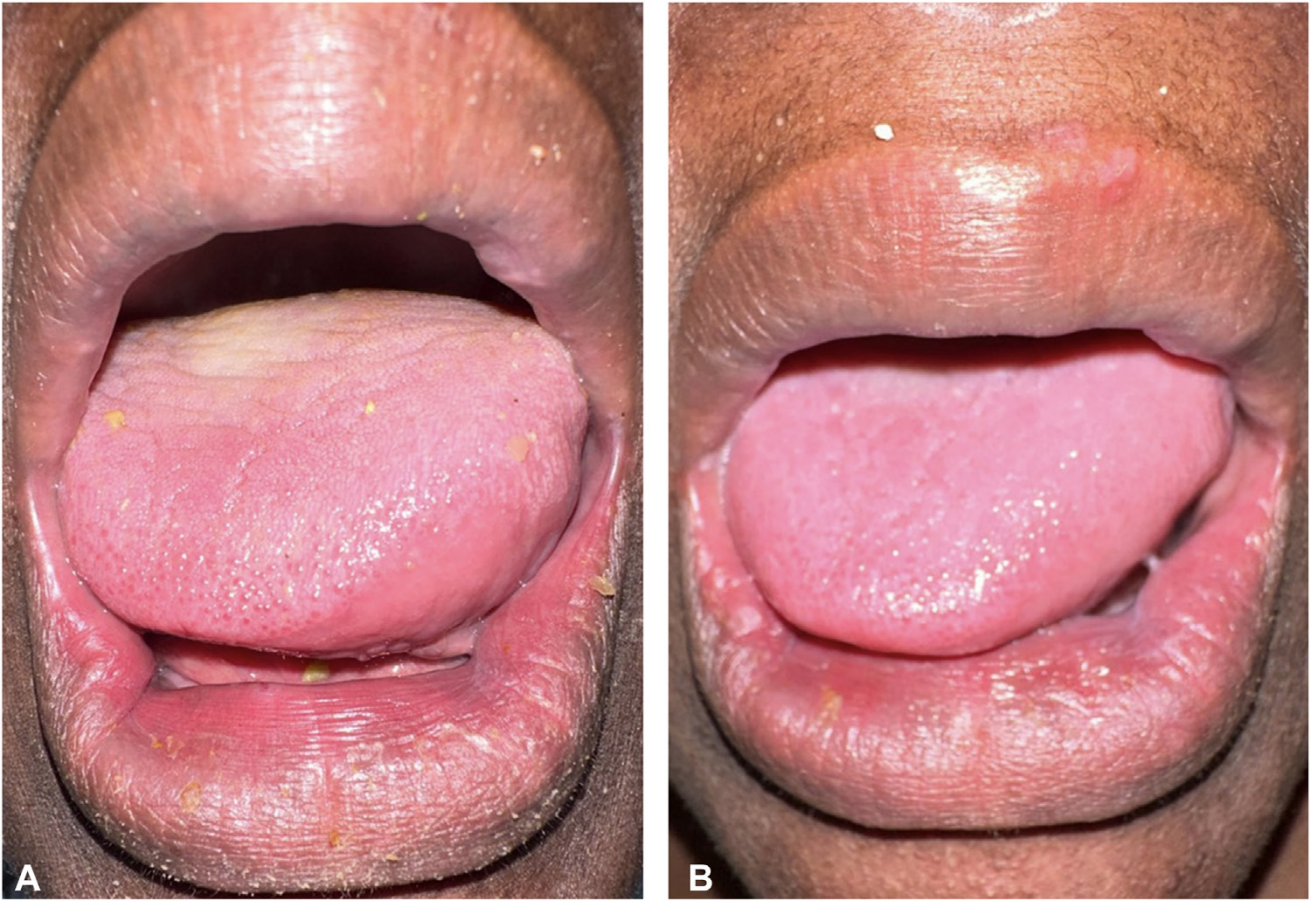
Improvement of macroglossia and lip edema with intravenous immunoglobulin therapy. **A,** Initial presentation showing macroglossia (without fissures) and edematous lips. **B,** Marked reduction in lip and macroglossia following first intravenous immunoglobulin infusion.

**Fig 3:**
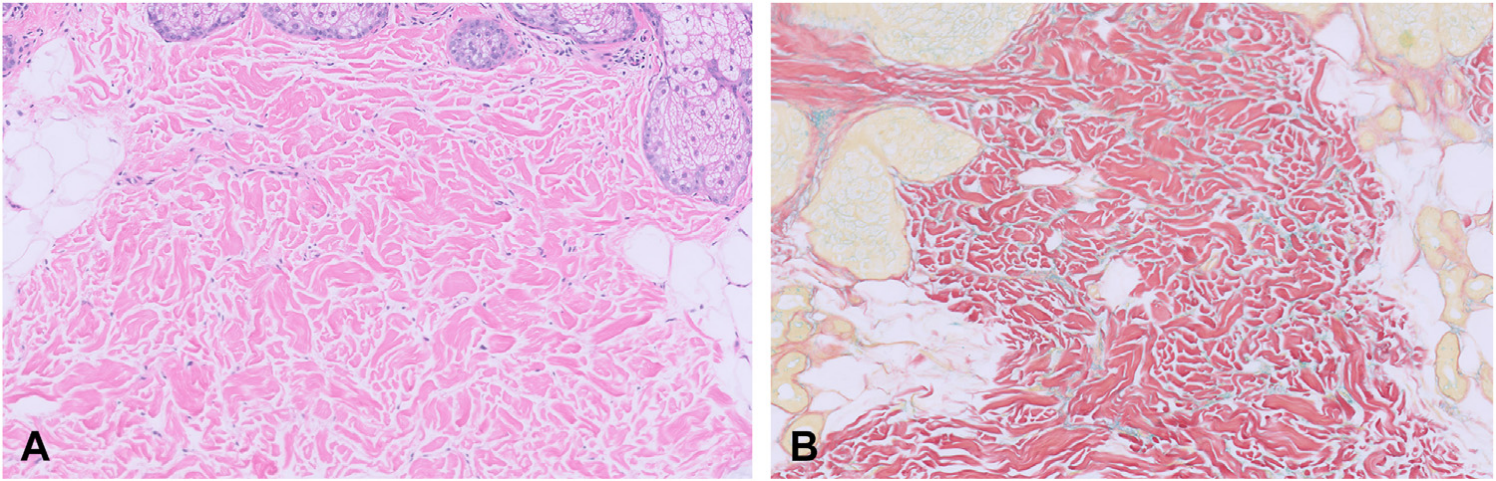
Punch biopsy of left cheek consistent with scleredema. **A,** (Hematoxylin and eosin, 10×) There is increased interstitial edema and subtle mucin deposition between the collagen bundles of the reticular dermis. **B,** (colloidal iron, 10×) A colloidal iron stain highlights interstitial mucin.

A 55-year-old woman with a history of seizures, hepatitis B, schizophrenia, and bipolar disorder was evaluated by the inpatient dermatology service for worsening generalized edema over the past few months. On skin exam, the cheeks, upper back, and proximal upper extremities felt firm (indurated plaques) without erythema or dyspigmentation; the cheeks were swollen compared to prior photos (Fig 1, *A*). Other significant findings were lip edema and macroglossia without oral papilloma, fissures, or ulcerations (Fig 2, *A*). Laboratory workup revealed IgG kappa monoclonal gammopathy with the following characteristics: IgG 1985 mg/dL (*N*: 700-1600), serum kappa/lambda free ratio 3.47 (*N*: 0.26-1.65), gamma M-spike 0.96 g/dL on serum protein electrophoresis. The urine protein electrophoresis was negative for paraproteins. Complete blood chemistry, basic metabolic panel, liver function test, antinuclear antibody, extractable nuclear antigen antibody, complements C3 and C4, IgG4 subtype, thyroid stimulating hormone, and hemoglobin A1C were all within normal limits. Computed tomography of the abdomen, pelvis, cervical spine, chest, and facial bones was unremarkable. A biopsy of the left cheek demonstrated subtle dermal collagen alteration with an increase in dermal mucin (Fig 3, *A*), highlighted with a colloidal iron stain (Fig 3, *B*). The constellation of clinicopathologic and serologic findings support the diagnosis of PAS.

Before dermatology’s evaluation, the patient was started on oral prednisolone (80 mg for 2 days followed by 70 mg for 2 days) but was stopped due to the lack of clinical improvement. Following the diagnosis of PAS, IVIg dosed at 2 g/kg administered over 2 days resulted in rapid improvement of the cheeks (Fig 1, *B*), tongue (Fig 2, *B*), upper neck, and back. At 1 month follow-up, the patient continued to show interval improvement in facial, oral, and truncal edema. The patient is scheduled for monthly IVIg infusion and hematology-oncology follow-up for further management of her paraproteinemia.

## Discussion

While extracutaneous manifestations of scleredema have been described, including buccal and parotid involvement, reports of tongue swelling are exceedingly rare.^2^^,^^3^ Ulmer et al^4^ published a case of scleredema with tongue, pharyngeal, and esophageal involvement in a 75-year-old male with IgG lambda monoclonal gammopathy and sicca syndrome. Scleredema with tongue swelling can mimic other dermatologic disorders, such as systemic amyloidosis and lipoid proteinosis, which typically present with small lingual papules or ulcerations.^5^^,^^6^

There is no consensus on a therapeutic protocol for the treatment of scleredema. Options for PAS include thalidomide, which has had limited success, particularly in the setting of IgA monoclonal gammopathy.^7^^,^^8^ One report showed successful treatment of PAS with IVIg after failing thalidomide.^8^ Our case further supports the use of IVIg in the management of PAS. Additionally, we recommend adding macroglossia and lip swelling as the initial presenting signs of PAS.

## Conflicts of interest

None disclosed.
